# Supplementary material for: Pepper Fruit Extracts Show Anti-Proliferative Activity against Tumor Cells Altering Their NADPH-Generating Dehydrogenase and Catalase Profiles
Source: Antioxidants (Basel). 2023 Jul 20;12(7):1461. doi: 10.3390/antiox12071461 (PMC10376568; doi:10.3390/antiox12071461)
Supplement: Supplementary file 1 [file antioxidants-12-01461-s001.zip › antioxidants-2488576-supplementary.pdf]

Supplementary Table S1. Tyrosine (Y) nitrated polypeptides from Hep-G2 and MIA PaCa-2 tumor cell lines after the incubation with pepper fruit crude extracts from the variety Alegría riojana. The number of pixels, determined by using the program ImageJ, is given in those polypeptides which were differentially expressed after the treatment of cells with the pepper fruit crude extracts.

Cell line	Tyrosine (Y) nitrated polypeptides	Size (kDa)	No. Pixels (control cells)	No. Pixels (treated cells)
Hep-G2	PHY 1	47.6		
	PHY 2	45.7		
	PHY 3	36.7		
	PHY 4	33.5	23734	15490
	PHY 5	25.0		
MIA PaCa-2	PMY 1	65.1	5407	4594
	PMY 2	47.6		
	PMY 3	45.7		
	PMY 4	36.7		
	PMY 5	33.0		
	PMY 6	29.5	7256	10351
	PMY 7	25.0	8333	12005
	PMY 8	18.4		
	PMY 9	16.4		

Supplementary Table S2. Tryptophan (W) nitrated polypeptides from Hep-G2 and MIA PaCa-2 tumor cell lines after the incubation with pepper fruit crude extracts from the variety Alegría riojana.

Cell line	Tryptophan (W) nitrated polypeptides	Size (kDa)
Hep-G2	PHW 1	65.3
	PHW 2	55.6
	PHW 3	45.5
	PHW 4	40.7
	PHW 5	37.0
	PHW 6	35.1
	PHW 7	25.0
	PHW 8	14.2
MIA PaCa-2	PMW 1	71.3
	PMW 2	48.0
	PMW 3	43.9
	PMW 4	38.8
	PMW 5	34.1
	PMW 6	29.9
	PMW 7	26.5
	PMW 8	17.8
	PMW 9	15.6

Supplementary Table S3. Cysteine (C) nitrosated polypeptides from Hep-G2 and MIA PaCa-2 tumor cell lines after the incubation with pepper fruit crude extracts from the variety Alegría riojana.

Cell line	Cysteine (C) nitrosated polypeptides	Size (kDa)
Hep-G2	PHC 1	88.4
	PHC 2	68.4
	PHC 3	62.4
	PHC 4	31.3
	PHC 5	29.0
	PHC 6	25.0
MIA PaCa-2	PMC 1	50.0
	PMC 2	37.6
	PMC 3	29.0
	PMC 4	27.8
	PMC 5	22.3

Supplementary Table S4. Glutathionylated polypeptides from Hep-G2 and MIA PaCa-2 tumor cell lines after the incubation with pepper fruit crude extracts from the variety Alegría riojana. The number of pixels, determined by using the program ImageJ, is given in those polypeptides which were differentially expressed after the treatment of cells with the pepper fruit crude extracts.

Cell line	Glutathionylated polypeptides	Size (kDa)	No. Pixels (control cells)	No. Pixels (treated cells)
Hep-G2	PHG 1	62.6		
	PHG 2	47.0		
	PHG 3	42.7		
	PHG 4	38.2		
	PHG 5	35.2		
	PHG 6	33		
	PHG 7	26.2		
	PHG 8	24.0		
	PHG 9	15.6		
MIA PaCa-2	PMG 1	61.0		
	PMG 2	37.0		
	PMG 3	33.1	3565	2294
	PMG 4	22.6		
	PMG 5	15.0		
	PMG 6	11.6	2487	1107
